# The potency of chitosan-based *Pinus merkusii* bark extract nanoparticles as anti-cancer on HeLa cell lines

**DOI:** 10.14202/vetworld.2019.1616-1623

**Published:** 2019-10-25

**Authors:** Annise Proboningrat, Amaq Fadholly, Regina Purnama Dewi Iskandar, Agung Budianto Achmad, Fedik Abdul Rantam, Sri Agus Sudjarwo

**Affiliations:** 1 Doctoral Student, Faculty of Veterinary Medicine, Universitas Airlangga, Surabaya 60115, Indonesia; 2 Doctoral Student, Faculty of Medicine, Universitas Airlangga, Surabaya 60115, Indonesia; 3 Department of Health, Faculty of Vocational Studies, Universitas Airlangga, Surabaya 60115, Indonesia; 4 Department of Microbiology, Faculty of Veterinary Medicine, Universitas Airlangga, Surabaya 60115, Indonesia; 5 Department of Pharmacology, Faculty of Veterinary Medicine, Universitas Airlangga, Surabaya 60115, Indonesia

**Keywords:** chitosan, HeLa cells, nanoparticles, *Pinus merkusii*

## Abstract

**Background and Aim:**

Cervical cancer accounts for the fourth as a cause of death from cancer in women worldwide, with more than 85% of events and deaths occurring in developing countries. The main problems of chemotherapy are the lack of selectivity and drug resistance. This study aimed to investigate the signal transduction of chitosan-based *Pinus merkusii* bark extract nanoparticles (Nano-PMBE) as an anticancer on HeLa cell line.

**Materials and Methods:**

Nano-PMBE was prepared based on the ionic gelation method. Its anticancer activities in HeLa cells were investigated through cytotoxicity test, cell cycle, and apoptosis analysis. The expression of p53 and caspase-9 was also observed.

**Results:**

The results showed that Nano-PMBE has a size of 394.3 nm. Meanwhile, the Nano-PMBE was cytotoxic to HeLa cells (IC_50_ of 384.10 µg/ml), caused G0/G1 phase arrest and cell apoptosis in HeLa cells. Besides, the expression of p53 and caspase-9 has increased.

**Conclusion:**

The results showed a notable anticancer effect of Nano-PMBE by arresting the cell cycle and inducing apoptosis in HeLa cells, suggesting that it might have therapeutic potential for cervical cancer. Further research is needed to find out more about the anticancer mechanism of Nano-PMBE in HeLa cells to *in vivo* and clinical studies.

## Introduction

The second most common cancer in the world is cervical cancer. It accounts for the fourth as a cause of death from cancer in women around the world, with more than 85% of events and deaths occurring in developing countries [1,2]. Most cervical cancer cells infected by the human papillomavirus are known to express E6 and E7 viral oncogenes, which suppress cellular regulatory proteins that play an important role in cell growth, differentiation, survival, apoptosis, and genome stability [3].

To date, chemotherapy is still the most commonly used cancer therapy method. However, chemotherapy still has some disadvantages due to lack of selectivity to cancer cells that lead to less concentration of drugs at the cancer site, systemic toxicity, and the emergence of drug-resistant cancer cells [4]. A breakthrough to overcome these various problems is by developing an anticancer agent in using nanoparticle technology [3]. Nanoparticles are particles that have a range of sizes between 1 and 1000 nm, which makes it has a wider surface area than microparticles. The small size of nanoparticle also allows easier administration of drugs [5] and the ability to penetrate barriers in the body, including the blood-brain barrier [6]. Besides, the cellular uptake mechanism of nanoparticles mainly through endocytosis allows it to enter specific cells and accumulate in them [7], thereby reducing the side effects of anticancer drugs [8]. Of all nano-based drug delivery approaches, polymeric-based nanoparticles offer very significant advantages and widely available formulation methods [9]. Chitosan is a form of carbohydrate polymer formed from N-deacetylation process of chitin, which is abundant in nature with good biocompatibility and biodegradability [10,11]. Chitosan is known to have a variety of pharmacological activities such as antibacterial [12], antifungal [13], anti-inflammatory [14], immunostimulant [15], and antitumor [10]. In addition, formulations of chitosan nanoparticles have been widely used to load and deliver drugs and vaccines, such as curcumin [13], mifepristone [11], ciprofloxacin [16], and ampicillin [17]. Natural therapies with herbal products have been carried out for many years in the treatment of cancer with very low side effects. It has been reported that the phytochemical content of plants could inhibit the proliferation of cancer cells directly [2]. *Pinus merkusii* is one of the original pine plants from Southeast Asia, including Indonesia [18]. The medical properties of pine species have shown excellent pharmacological effects, including anticancer activities. Experimental studies report that tree bark of the pine species has the main content of proanthocyanidins [19] that show anticancer activity through the mechanism of apoptosis-related to the increased of pro-apoptotic protein expression [20], Bax; decreased expression of the anti-apoptotic protein, Bcl-2; and Caspase 9 and 3 activation [21,22]. In addition, one species of the pine plants have also been shown to cause inhibition of the cancer cell cycle throughcyclin-dependent kinase (CDK) 1 and cyclin B downregulation mechanisms and upregulation of p53 and p21 activity in human liver cancer HepG2 cells [22].

This study aimed to investigate the signal transduction of chitosan-based *P*. *merkusii* bark extract nanoparticles (Nano-PMBE) as an anticancer agent on human cervical cancer HeLa cell lines.

## Materials and Methods

### Ethical approval

The current study was approved by Medical and Health Research Ethics Committee, Faculty of Medicine, Universitas Gadjah Mada, Indonesia (approval number 3.1-007.2013.03).

### Chemicals

Chitosan (50,000-190,000 Da, Sigma-Aldrich, USA), glacial acetic acid (100%, 60.05 g/mol, Merck, Germany), sodium tripolyphosphate (NaTPP) (367.86 g/mol, Sigma-Aldrich, USA), dimethyl sulfoxide (DMSO) (78.13 g/mol, Sigma-Aldrich, USA), Dulbecco’s modified eagle medium (Gibco, USA), hepes (238.30 g/mol, Sigma-Aldrich, USA), 10% fetal bovine serum (Rocky Mountain Biologicals, Inc., USA), 2% penicillin-streptomycin (Gibco, USA), 0.5% fungizone (924.08 g/mol, Sigma-Aldrich, USA), 1× phosphate buffer saline (PBS) (pH 7.4, Sigma-Aldrich, USA), trypsin-ethylenediaminetetraacetic acid solution (1×, pH 7.0-7.6, Sigma-Aldrich, USA), 3-(4,5-dimethylthiazol-2yl)-2,5-diphenyltetrazolium bromide (MTT) (414.32 g/mol, Sigma-Aldrich, USA), sodium dodecyl sulfate (288.38 g/mol, Sigma-Aldrich, USA), 0.1 N hydrogen chloride solution (34.46 g/mol, Sigma-Aldrich, USA), ribonuclease (RNAse) (BD Bioscience, CA), propidium iodide (PI) (668.39 g/mol, Sigma-Aldrich, USA), Triton^®^ X-100 (pro GC-Merck), Annexin V-FITC apoptosis detection kit with PI (BioLegend Inc., UK), hydrogen peroxide solution (H_2_O_2_) (1-5%, 34.01 g/mol, Sigma-Aldrich, USA), 1:200 p53 mouse monoclonal antibody (53 kDa, p53 Ab-1, Thermo Scientific™ Lab Vision™), 1:50 anti-caspase-9 antibody (ab52298, Abcam), Star Trek Universal Horseradish Peroxidase (HRP) Detection System (Biocare Medical, USA), Mayer’s hematoxylin solution (Sigma-Aldrich, USA), Entellan® (Merck), methanol (absolute, 32.04 g/mol Sigma-Aldrich, USA), ethanol (≥99.8%, 46.07 g/mol, Sigma-Aldrich, USA), and distilled water.

### Preparation of *P. merkusii* bark extract

Stem barks of *P*. *merkusii* were collected from Malang, Indonesia. The plant specimen was compared to herbarium collection deposited at Purwodadi Botanical Garden, Indonesian Institute of Sciences, Indonesia. The dry *P*. *merkusii* barks were cut into pieces and ground into powder. 350 g of the powdered bark was soaked in 96% ethanol (1.75 L) for 3 days, and then the macerate was separated and concentrated using a rotary evaporator (250 rpm, 60°C).

### Preparation of Nano-PMBE loaded chitosan nanoparticles

Nano-PMBE loaded in chitosan nanoparticles was prepared based on the ionotropic gelation method [23]. 0.2 g of chitosan was dissolved in 1% acetic acid solution. 1 g of the ethanolic extract of *P*. *merkusii* bark was dissolved in 35 ml of ethanol and added with 15 ml of distilled water. Then, the suspension was added to the 0.2% chitosan solution. Furthermore, 350 ml of 0.84% NaTPP solution was dripped onto the chitosan solution slowly with magnetic stirring at 1000 rpm for approximately 2 h. The comparative composition of chitosan and NaTPP used was 8:1. The nanoparticle suspension was separated between the precipitate and the colloid. The nanoparticle colloid was freeze-dried, and the obtained nanoparticle powder was then dissolved in 2% DMSO for culture cell treatment.

### Particle size analysis

Dynamic light scattering method was performed to measure the size of the Nano-PMBE and polydispersity index (PDI) using Zetasizer Nano ZS (Malvern Instrument Ltd., UK).

### Cell culture

HeLa cells were obtained from the Department of Parasitology (Faculty of Medicine, Gadjah Mada University, Indonesia) and were cultured in Dulbecco’s modified eagle medium (Gibco, USA) equipped with 10% fetal bovine serum (Rocky Mountain Biologicals, Inc., USA), 0.5% fungizone (924.08 g/mol, Sigma-Aldrich, USA), and 2% penicillin-streptomycin (Gibco, USA) at 37°C in a 5% CO_2_ incubator.

### Experimental procedure

The experiment was started with the preparation of chitosan-based nanoparticles loading Nano-PMBE. The synthesized Nano-PMBE was characterized to find out the particle size and distribution. Then, it was divided into five concentrations (50, 100, 200, 400, and 800 µg/ml in DMSO) for MTT cytotoxicity assay and to determine the IC_50_ value. Based on the IC_50_ value, Nano-PMBE was then divided into three groups of concentration, including IC_50_, 2IC_50_, and 4IC_50_, and the obtained values were 384.10, 768.2, and 1536.4 µg/ml, respectively. Subsequently, these three concentrations were used for analyzing cell cycle arrest, apoptosis, and expression of p53 and caspase-9 protein of HeLa cell lines.

### Determination of IC_50_ value

The IC_50_ value of Nano-PMBE was determined by the MTT assay method [2]. HeLa cells (1×10^4^ cells/well) were seeded in 96-well plates overnight. Then, they were treated with a concentration series of Nano-PMBE starting with 800 µg/ml as the highest concentration (fourfold dilution) for 24 h incubation. 100 µl of MTT (414.32 g/mol, Sigma-Aldrich, USA) solution was added, incubated for 4 h followed by 100 µl of 10% sodium dodecyl sulfate (288.38 g/mol, Sigma-Aldrich, USA). The absorbance of formazan was measured by Benchmark Microplate Reader (Bio-Rad, USA) at 595 nm. Then, the inhibition percentage was calculated and used to determine the IC_50_ value employing linear regression analysis using Microsoft Excel 2016.

### Cell cycle analysis

HeLa cells (1×10^6^ cells/well) were seeded in 6-well plates. After overnight incubation (37°C, 5% CO_2_), the cells were treated with Nano-PMBE (384.10, 768.2, and 1536.4 µg/ml) for 24 h. They were then centrifuged at 2500 rpm for 10 min. About 70% cold ethanol was used for fixation. Then, 400 µl of PI (668.39 g/mol, Sigma-Aldrich, USA) and 100 µl of RNAse (BD Bioscience, San Diego, CA) were added and incubated for 30 min in the dark, at room temperature (25°C). The cells were analyzed using BD FACSCalibur flow cytometer (BD Biosciences, USA) to determine the cell population in each cell cycle phase.

### Apoptosis analysis by Annexin V-FITC/PI assay

Apoptosis of the cells was detected by Annexin V/PI staining method using FITC Annexin V apoptosis detection kit with PI (BioLegend Inc., UK). HeLa cells were plated in 6-well plates (1×10^6^ cells/well) and incubated overnight (37°C, 5% CO_2_). Then, they were treated with 384.10, 768.2, and 1536.4 µg/ml of Nano-PMBE for 24 h. After being harvested, the cells were washed twice with cold 1× PBS (pH 7.4, Sigma-Aldrich, USA) and resuspended in Annexin V binding buffer (0.25-1.0×10^7^ cells/ml, BioLegend Inc., UK), then they were stained with 5 µl of Annexin V and 10 µl of PI (BioLegend Inc., UK) and incubated at room temperature (25°C) for 15 min in the dark. Finally, the cells were analyzed using BD FACSCalibur flow cytometer (BD Biosciences, USA).

### Immunocytochemistry staining

Briefly, HeLa cells (2×10^4^ cells/well) were grown on plastic coverslips inside 24-well plates and incubated at 37°C with 5% CO_2_, before being treated with 384.10, 768.2, and 1536.4 µg/ml of Nano-PMBE for 24 h. Then, they were fixed in 500 µl of methanol (absolute, 32.04 g/mol Sigma-Aldrich, USA) for 10 min, washed with 1× PBS (pH 7.4, Sigma-Aldrich, USA), and treated with 1 ml of 1% hydrogen peroxide solution (1-5%, 34.01 g/mol, Sigma-Aldrich, USA) in absolute methanol (1:10) for 10 min. Subsequently, the cells were blocked with background sniper followed by staining with 1:200 p53 mouse monoclonal antibody (53 kDa, p53 Ab-1, Thermo Scientific™ Lab Vision™) and 1:50 anti-caspase-9 antibody (ab52298, Abcam) for 1 h in the dark. The stained cells then were washed with 1× PBS (pH 7.4, Sigma-Aldrich, USA) twice. The protein expression was detected by the Starr Trek Universal HRP Detection System. Counterstaining of the cells was done using Mayer’s hematoxylin solution (Sigma-Aldrich, USA), and the cells dipped in ethanol (≥99.8%, 46.07 g/mol, Sigma-Aldrich, USA) and dried. Then, the coverslips were mounted onto glass slides and visualized using Nikon Eclipse Ci microscope (Nikon Corp., Tokyo, Japan). The expression of p53 and caspase-9 was evaluated semi-quantitatively based on H-score method [24].

### Statistical analysis

Results were analyzed by regression analysis using Microsoft Excel 2016, by one-way analysis of variance and Kruskal–Wallis using IBM Statistics SPSS 25. p≤0.05 was considered to be significant.

## Results

### The particle size of Nano-PMBE

**Figure-1:**
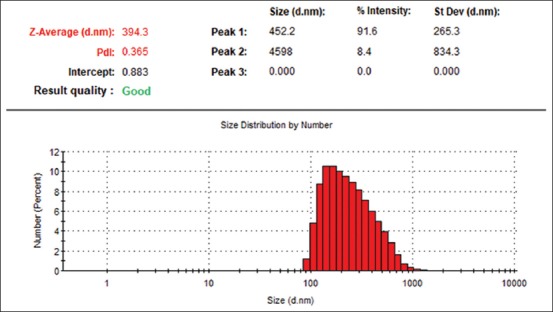
Particle size distribution of synthesized Nano-PMBE.

The result of particle size analysis shows that the average size (Z-average) of Nano-PMBE is 394.3 nm with a good PDI, 0.365 (Figure-1).

### The IC_50_ value of Nano-PMBE on HeLa cells

**Figure-2:**
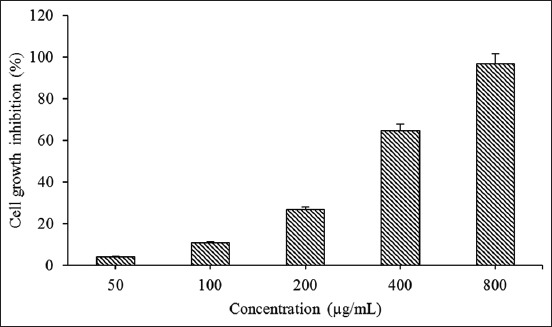
The effects of Nano-PMBE on HeLa cells. Inhibition of the cell growth was assessed by 3-(4,5-dimethylthiazol-2yl)-2,5-diphenyltetrazolium bromide assay.

MTT assay [2] was employed to find out the capability of Nano-PMBE to inhibit the growth of HeLa cells and to determine the IC_50_ value. MTT test was carried out on HeLa cells treated with a range of concentrations of Nano-PMBE (50, 100, 200, 400, and 800 µg/ml). Nano-PMBE shows an elevated ability to inhibit cell growth, dose-dependently (Figure-2), with an IC_50_ value of 384.10 µg/ml after 24 h incubation.

### Cell cycle arrest of HeLa cells induced by Nano-PMBE

**Figure-3:**
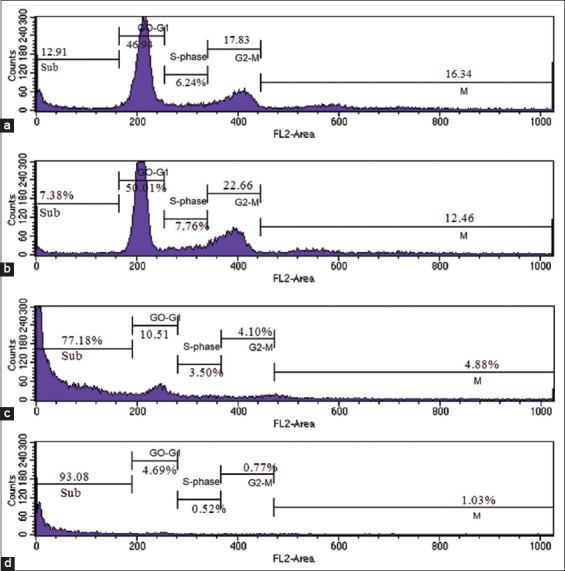
Effects of Nano-PMBE on cell cycle distribution in HeLa cells for 24 h detected by flow cytometric analysis, (a) control, (b) 384.10 µg/ml, (c) 768.2 µg/ml, and (d) 1536.4 µg/ml.

As shown in Figure-3, there is a significant G0/G1 phase arrest in HeLa cells treated with Nano-PMBE. After 24 h of treatment, the population of HeLa cells at G0/G1 phase significantly increased from 46.94% (control) to 50.01% (384.10 µg/ml). Subsequently, Nano-PMBE at 768.2 µg/ml and 1536.4 µg/ml increased 77.18% and 93.08% cell population in sub G1 phase (hypodiploid DNA) which is considered in apoptosis state. However, the cell population in S phase is dose-dependently decreased, and treatment with Nano-PMBE did not show significant effects on G2/M phase. The results of cell cycle analysis indicated that Nano-PMBE induces G1 phase arrest and apoptosis (sub G1) as the concentration increased.

### Apoptosis of HeLa cells induced by Nano-PMBE

**Figure-4:**
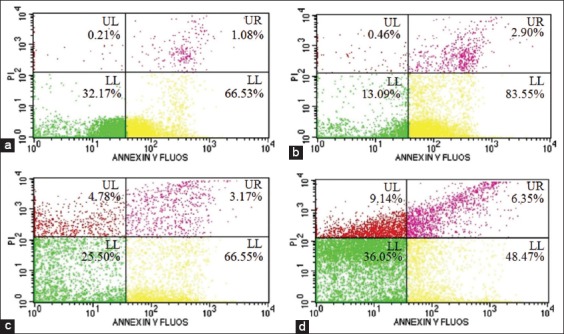
Flow cytometric analysis of apoptosis induction in HeLa cells treated with Nano-PMBE for 24 h, (a) control, (b) 384.10 µg/ml, (c) 768.2 µg/ml, and (d) 1536.4 µg/ml. The histogram in the lower left, lower right, upper right, and upper left quadrant represented as viable, early apoptotic, late apoptotic, and necrotic cells, respectively.

To examine whether the effect of Nano-PMBE treatment is correlated to programmed cell death, Annexin V/PI staining was used to measure the number of apoptotic cells in HeLa cells. The apoptotic cells are represented by the percentage of early (Annexin V+/PI) and late apoptotic cells (Annexin V+/PI+) in the histogram. The HeLa cells undergo early apoptosis are elevated from 66.53% (control) to 83.55% after treated with 384.10 µg/ml of Nano-PMBE for 24 h; however, the early apoptotic cells are decreased in treatment with 768.2 µg/ml and 1536.4 µg/ml of Nano-PMBE. Meanwhile, the percentage of late apoptotic cells is elevated dose-dependently from 1.08%, 2.90%, 3.17%, and 6.35% of control cells to 384.10 µg/ml, 768.2 µg/ml, and 1536.4 µg/ml of Nano-PMBE, respectively. However, they are significantly lower than the percentage of early apoptotic cells. Subsequently, the percentage of cells at necrosis state is elevated dose-dependently (Figure-4). It suggested that the treatment of Nano-PMBE with an incubation period of 24 h tends to induce early apoptosis in HeLa cells.

### p53 expression of HeLa cells induced by PMBE nanoparticles

**Figure-5:**
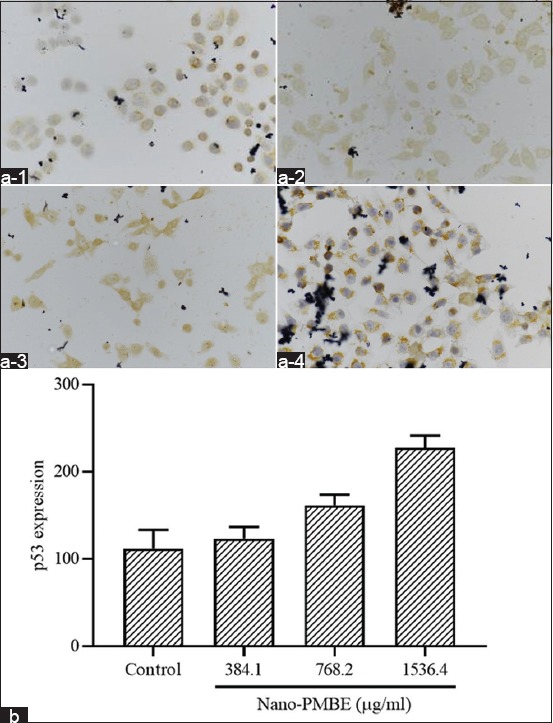
Microscopical observation of p53 expression in (a-1) untreated HeLa cells and HeLa cells treated with (a-2) 384.10 µg/ml, (a-3) 768.2 µg/ml, and (a-4) 1536.4 µg/ml of Nano-PMBE for 24 h (Nikon Eclipse Ci microscope; magnification 400×). (b) Bar represents mean±standard deviation. Of the expression score of p53 in treated HeLa cells.

To verify whether PMBE-induced G0/G1 phase arrest in HeLa cells was relevant to p53 activation, immunocytochemistry was performed to evaluate the p53 expression level. The protein expression was calculated semi-quantitatively based on H-score method [24]. After 24 h of treatment with 384.10 µg/ml of Nano-PMBE, the p53 expression is elevated in HeLa cells compared to control. Subsequently, the p53 expression is gradually decreased in HeLa cells treated with 768.2 µg/ml and 1536.4 µg/ml of Nano-PMBE (Figure-5). The present finding implies that PMBE nanoparticles promote the expression of p53 protein in HeLa cells.

### Caspase-9 expression of HeLa cells induced by PMBE nanoparticles

**Figure-6:**
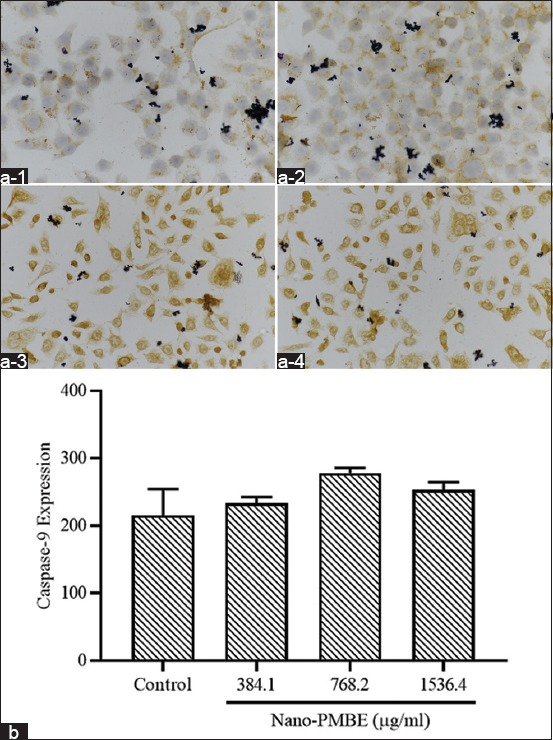
Microscopical observation of caspase-9 expression in (a-1) untreated HeLa cells and HeLa cells treated with (a-2) 384.10 µg/ml, (a-3) 768.2 µg/ml, and (a-4) 1536.4 µg/ml of Nano-PMBE for 24 h (Nikon Eclipse Ci microscope; magnification 400×). (b) Bar represents mean±standard deviation of the expression score of caspase-9 in treated HeLa cells.

The expression of caspase-9 in HeLa cells following Nano-PMBE treatment was examined by immunocytochemistry staining and calculated by the H-score method [24]. The expression of caspase-9 is elevated dose-dependently in HeLa cells treated with 384.10 µg/ml and 768.2 µg/ml of Nano-PMBE compared to control. Subsequently, it decreased in HeLa cells treated with 1536.4 µg/ml of Nano-PMBE (Figure-6). The results demonstrate that Nano-PMBE induces apoptosis in HeLa cells through the elevation of caspase-9 expression.

## Discussion

In this study, a novel chitosan nanoparticle was formulated to load Nano-PMBE to investigate its signal transduction as an anticancer agent in human cervical cancer, HeLa cell line. Briefly, MTT assay was conducted to determine the cytotoxicity and IC_50_ value of Nano-PMBE. The treated cells were then analyzed by flow cytometry and Annexin V-FITC/PI assay to investigate the cell cycle arrest and apoptosis induction by Nano-PMBE. Finally, immunocytochemistry staining was conducted to evaluate the expression of p53 and caspase-9 induced by Nano-PMBE in HeLa cells.

The results indicate that Nano-PMBE induces an antiproliferative effect on cervical cancer cells, dose-dependently, through the MTT assay. Previously, it has been reported that tree bark of other pine species induces cytotoxicity in HeLa cells [20] and other cancer cells, such as MCF-7 [25], A2780 ovarian cancer cells [21], and IMR-32 neuroblastoma cancer cells [26].

Cancer is commonly known as a dysfunction disease of the cell cycle. Molecular dysregulation of the cell cycle is one of the most crucial changes during tumor progression [27]. Therefore, the cell cycle is one of the effective targets for cancer therapy [28]. To find out whether induction of growth inhibition by Nano-PMBA is mediated by cell cycle arrest, we evaluated the distribution of cell cycles of HeLa cancer cells using flow cytometry. This study shows that Nano-PMBA inhibits the cell cycle of HeLa cells in the G0/G1 phase. Inhibition of the cell cycle is a checkpoint mechanism in cell cycle progression initiated by p53 tumor suppressor [29,30]. When there is a cellular stress signal, the transcription factor of p53 will be activated by Chk2. p53 then induces the synthesis of a p21 protein, which can inhibit CDK2 and cyclin E activity. Thus, the cell cycle can be inhibited in the G1 phase [30,31]. In this study, there was an increase in the expression of p53 protein in HeLa cells treated with Nano-PMBE, thus confirming that the cell cycle inhibition in the G0/G1 phase was mediated by p53. These results are in line with previous studies which reported that pine plants could inhibit the liver cancer cell cycle of HepG2 by suppressing Cyclin B and CDK1 and increasing expression of p53 and p21 [22].

Apoptosis is another promising approach to cancer therapy [30]. Apoptosis has a very significant role in eliminating tumor cells that have mutated or overgrown. Several types of herbal medicines have been reported to inhibit cancer cell growth through the induction of apoptosis [21]. The results of this study showed that Nano-PMBE significantly induces apoptosis in HeLa cells. These results were further confirmed by the increased expression of caspase-9, an initiator caspase protein in the event of programmed cell death, in HeLa cells treated with Nano-PMBE.

Apoptosis induction is associated with down- and up-regulation of anti-apoptotic and pro-apoptotic proteins, respectively. It has been known that Bcl-2 plays a major role in the regulation of the intrinsic apoptosis pathway. Suppression of Bcl-2 resulting in increased permeability of the mitochondrial membrane, releasing cytochrome c into cytoplasm and activating caspase-9. The activated caspase-9 then activates caspase-3, the executor caspase, to induce apoptotic events [21,27]. Pine plants are well known to have a rich content of proanthocyanidins [19-21]. Previous studies have reported that proanthocyanidin induces apoptosis in cervical cancer cells by suppressing the expression of the Bcl-2 protein, increasing Bax expression, and activating caspases 9 and 3 [20].

## Conclusion

Nano-PMBE exerts an inhibitory effect on the growth of cervical cancer cells through inducing G0/G1 phase arrest and apoptosis. A regulatory protein related to cell cycle arrest, p53, and an apoptotic caspase protein, caspase-9, were also upregulated by Nano-PMBE. Therefore, Nano-PMBE might have therapeutic potential for cervical cancer and is interesting for further studies to reveal its underlying mechanisms in cervical cancer cell therapy. *In vivo* and clinical studies need to be done to confirm the efficacy of these plant nanoparticles as an anticancer agent.

## Authors’ Contributions

AP, AF, RPDI, and SAS designed and performed the experiments. AP, ABA, FAR, and SAS interpreted the data and drafted themanuscript. AP and SAS revised the manuscript. All authors read and approved the final manuscript.
